# A Novel Platelet Activating Factor Receptor Antagonist Reduces Cell Infiltration and Expression of Inflammatory Mediators in Mice Exposed to Desiccating Conditions after PRK

**DOI:** 10.1155/2009/138513

**Published:** 2009-12-09

**Authors:** Salomon Esquenazi, Jiucheng He, Na Li, Nicolas G. Bazan, Isi Esquenazi, Haydee E. P. Bazan

**Affiliations:** LSU Eye Center and Neuroscience Center, LSU Health Sciences Center, School of Medicine, New Orleans, LA 70112, USA

## Abstract

*Purpose*. To study the contribution of a novel PAF receptor antagonist LAU-0901 in the modulation of the increased inflammatory response in mice exposed to dessicating conditions (DE) after PRK. *Methods*. Eighty 13-14 week old female Balb/C mice were used. They were divided into two groups: One group was treated with LAU-0901 topical drops. The other group was treated with vehicle. In each group ten mice served as controls and ten were placed in DE. The other twenty mice underwent bilateral PRK and were divided in two additional groups: ten mice remained under normal conditions (NC) and the other ten were exposed to DE. After 1 week all animals underwent in vivo confocal microscopy, immunostaining and western blotting analysis. *Results*. Confocal microscopy showed an increased number of reflective structures in the corneal epithelium after PRK and exposure to DE in eyes treated with vehicle as compared to eyes treated with LAU-090). Significant decrease of COX-2 and Arginase I expression and reduced alpha SMA cells was observed after PRK and exposure to DE in eyes treated with LAU-0901. Discussion: Exposure of mice to a DE after PRK increases the epithelial turnover rate. PAF is involved in the inflammatory cell infiltration and expression of inflammatory cytokines that follow PRK under DE.

## 1. Introduction

Following PRK, the corneal healing process is initiated after the epithelial injury through the release of multiple cytokines and growth factors such as IL-1, TNF*α*, EGF, and PDGF. The initial insult induces keratocyte apoptosis and necrosis [1]. Myofibroblasts appear in the subepithelial stroma 1-2 weeks after the injury or surgery, presumably derived from keratocytes under the influence of TGF-*β* [2]. Abundant inflammatory cells including monocytes, granulocytes, and lymphocytes begin to appear in the corneal stroma soon after an injury or surgery [3]. Previous studies have shown that PAF is a potent inflammatory mediator in the cornea that acts through specific receptors and stimulates cell infiltration and expression of cyclooxygenaese-2 (COX-2) [1]. We have demonstrated that there is an increase in inflammatory cell infiltration and COX-2 expression in mice corneas exposed to desiccating environment (DE) following PRK [4]. Possible contributions to the wound healing process are yet unknown; however, clinical studies have shown that dry eye conditions after LASIK produce long-term regression of the refractive results [5]. The present work studies the contribution of a novel PAF receptor antagonist LAU-0901 in the modulation of the increased inflammatory response in mice exposed to DE after PRK.

## 2. Methods

### 2.1. Animal Model

Eighty 13-14-week-old female Balb/C mice were used. Before surgery or in vivo confocal evaluation they were all examined using slit-lamp and fluorescein to assess the ocular surface integrity. The animals were treated according to the Resolution of human use of animals in Vision Research approved by the Association for Research in Vision and Ophthalmology, and the experimental protocol was approved by the institutional animal care and use committee, Louisiana State University Health Sciences Center. Animals were anesthetized intramuscularly with 2 mg/kg body weight of Xylazine and 50 mg/kg body weight of ketamine. They were divided in two groups. One group was treated with LAU-0901 topical drops 4 times a day for 1 week. The other group was treated with vehicle. From each group ten mice served as controls and ten were placed in DE created by placing the animals between two fans to obtain a continuous airflow of 15 L/min, in a room at 22°C with a relative humidity of 25%. Topical atropine 1% was applied twice a week for 2 weeks. The other twenty mice underwent bilateral corneal scraping using an electric brush (Algerbrush II, Alger Co, Lago Vista CA) involving the entire cornea without compromising the limbal area.. The animals were then divided in two additional groups: ten mice were placed in normal conditions (NC) and the other ten were exposed to DE.

### 2.2. In Vivo Confocal Microscopy

A Heidelberg retina tomography (HRT) II/Rostock Corneal Module (Heidelberg Engineering GmbH, Heidelberg, Germany) was used to examine the animals. Mice were anesthetized as explained previously and placed in a modified 50 mL centrifugation tubes mounted on a test tube holder as described earlier [4]. The HRT II camera was left connected to the head rest in a horizontal position. The laser source was a diode laser with a wavelength of 670 nm and the objective of the microscope is an immersion lens, magnification x60, numerical aperture 0.90 (Olympus, Hamburg, Germany). A drop of genteal gel (Novartis, St. Louis, MO) was placed on the tip of the objective lens to maintain immersion contact between the objective lens and the eye. Images covering an area of 400 × 400 *μ*m with transverse optical resolution of approximately 1 *μ*m/pixel were taken. Oblique sections of the cornea were obtained by controlling manually the *x*-*y* position and the depth of the optical section. For all eyes 20 confocal microscopy images of each layer including the superficial and basal epithelium, anterior and posterior stroma and endothelium were recorded. The images were then analyzed qualitatively and quantitatively and compared between the two groups.

### 2.3. Quantification of Cells and Nerves

Superficial and basal epithelial, anterior and posterior stromal and endothelial cell densities were measured using the program associated with the HRT II/RCM as described earlier [4]. Finally, the number of marks was counted by the computer and cellular densities were expressed as cells per mm^2^. The results were collected in a computer spread sheet (Excel 2000; Microsoft Corp., Redmond, WA). Statistical differences were calculated using the Statistical Program for Social Sciences (SPSS for Windows, ver 9.0; SPSS Sciences, Chicago, IL).

### 2.4. Immunofluorescence Staining

The mice were humanely euthanized and the eyes were immediately enucleated. Cryostat sections 8 *μ*m were prepared from each eyeball. After fixing, the sections were incubated with CD45, GR-1, CD 11b CD-11c, COX-2, Arginase II, and alpha-SMA antibodies overnight at 4°C. The sections were incubated with goat antirabbit IgG FITC-conjugated secondary antibody for 1 hour at room temperature. DAPI staining was performed to localize the nuclei. Sections were examined with a fluorescence microscope, and images were recorded with a digital camera.

### 2.5. Western Blot Analysis

Corneas were isolated and digested with 0.3% collagenease A to obtain the cells. Cells were homogenized in 50 mM Tris-HCl (pH 7.5), 1 mM EDTA, 1 mM EGTA, 0.5 mM sodium orthovanadate, 1 mM dithiothreitol (DTT), 1% Triton X-100, 5 mM sodium fluoride, 1 mM sodium pyrophosphate, 150 mM NaCl, 10 mM sodium *β*-glycerophosphate, 1 mM phenylmethylsulfonyl fluoride (PMSF), 1 *μ*g/mL aprotinin and leupeptin, and 1 *μ*M microcystin (lysis buffer). The homogenate was centrifuged at 12 000 rpm for 15 minutes, and total protein was determined as the supernatant. All procedures were performed at 4°C. Samples were resolved by SDS-polyacrylamide gel electrophoresis (9%–12% gel) and transferred to polyvinylidene difluoride (PVDF) membranes (Amersham Pharmacia Biotech, Piscataway, NJ). Biotinylated protein molecular weight standards were applied in one lane of each gel. The nonspecific proteins were blocked with 5% nonfat milk in Tris-buffered saline (TBS, 20 mM Tris-HCl, 150 mM NaCl [pH 7.6]) plus 0.1% Tween-20 for 1 hour and then probed with various primary antibodies, as described in the experiments for 2 hours at room temperature or overnight at 4°C. The membranes were washed six times with TBS plus 0.1% Tween-20 and further incubated with HRP-conjugated secondary antibodies. Protein bands were visualized using chemiluminescence detection reagents (ECL Plus; Amersham) and exposed to FUJIFILM LAS- 3000 system.

## 3. Results

### 3.1. In Vivo Confocal Microscopy

Images of corneal epithelium in mice after PRK and exposure to desiccating environment with and without treatment with LAU-0901 were analyzed using in vivo confocal microscopy method described previously [4]. Superficial epithelial cells showed a polygonal shape with hyperreflective nuclei surrounded by a hyporeflective area. The cytoplasm was more hyperreflective in the DE eyes compared with the NC corneas. 

**Figure 1:**
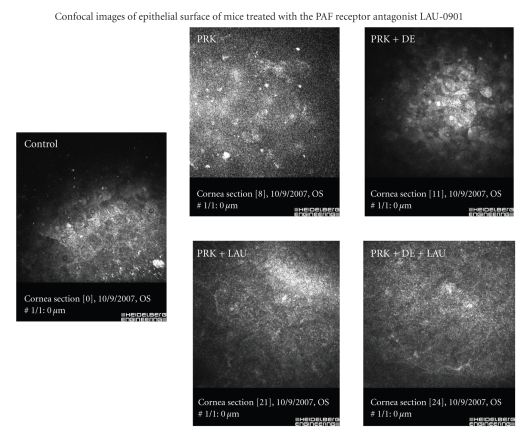
In vivo confocal microscopy images of corneal epithelium in mice after PRK and exposure to desiccating environment with and without treatment with LAU-0901. Increased number of reflective structures was observed in the corneal epithelium after PRK and exposure to DE in eyes treated with vehicle as compared with eyes treated with LAU-0901 (743 ± 128 cells/mm² versus 421 ± 109 cells/mm²). The increase in the number of reflective bodies was more significant in the vehicle group compared with LAU-0901 treated eyes.

 Increased number of reflective structures was observed in the corneal epithelium after PRK and exposure to DE in eyes treated with vehicle as compared with eyes treated with LAU-0901 (743 ± 128 cells/mm^2^ versus 421 ± 109 cells/mm^2^) which was statistically significant (Mann-Whitney U test; *P* = .05) (Figure 1). Basal cells appeared as dark cells with hyperreflective boundaries smaller than superficial cells and very closely organized. Its density was 746 ± 176 cells/mm^2^ in controls, 886 ± 168 cells/mm^2^ after PRK and DE in eyes treated with LAU-0901 and 1498 ± 293 cells/mm^2^ in the PRK and DE group treated with vehicle. There was a statistically significant increase in the cell count in the group treated with vehicle compared with LAU-0901 (Mann-Whitney U test; *P* < .05).

### 3.2. Immunostainning Studies

**Figure 2:**
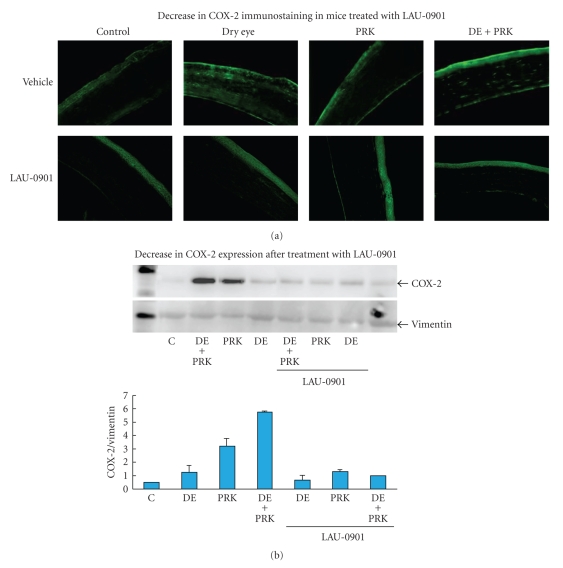
Significant decrease of COX-2 expression one week after PRK and exposure to desiccating conditions (DEs) in eyes treated with LAU-0901.

Significant decrease of COX-2 expression after 1 week of PRK and exposure to desiccating conditions in eyes treated with LAU-0901 was observed compared with non treated eyes and controls (Figure 2). 

**Figure 3:**
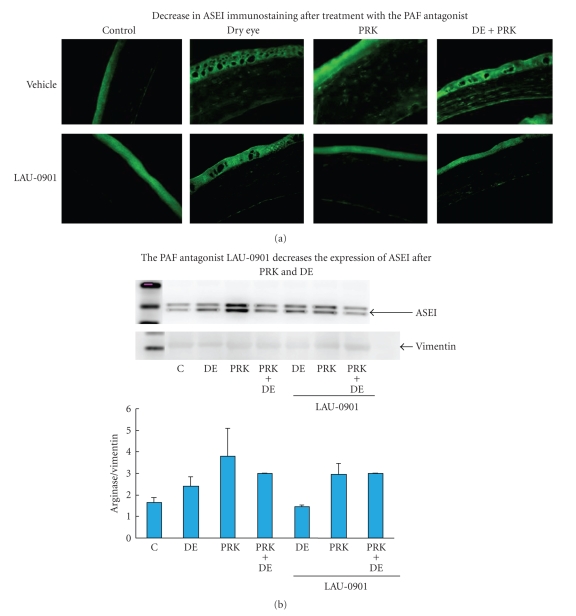
Significant decrease of Arginase I expression in mice corneas 1 week after PRK and exposure to desiccating conditions after treatment with LAU-0901.

Additionally, significant decrease of Arginase I expression in mice corneas 1 week after PRK and exposure to desiccating conditions after treatment with LAU-0901 was observed (Figure 3). 

**Figure 4:**
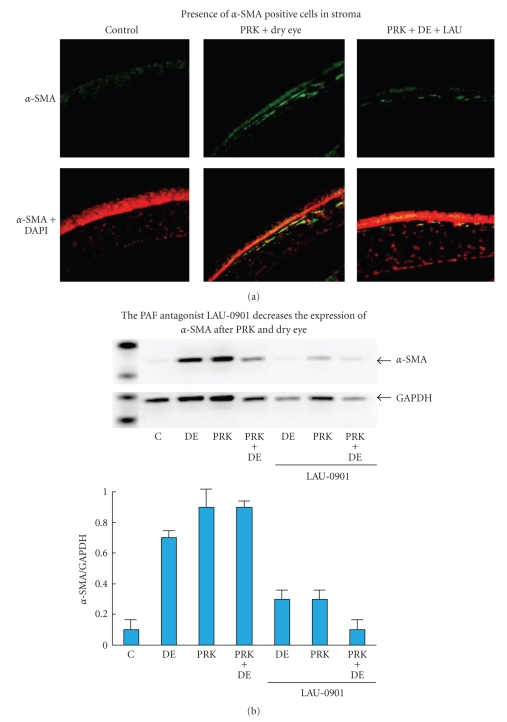
Reduced expression of alpha-SMA cells in mice corneas after PRK and exposure to desiccating environment and treatment with LAU-0901, one week postoperatively COX-2 after PRK and Dry Eye in mice corneas.

Analysis of alpha-SMA positive cells expression showed significant reduction in mice corneas after PRK and exposure to desiccating environment and treatment with LAU-0901, one week postoperatively (Figure 4).

## 4. Discussion

Exposure of mice to a desiccating environment after PRK increases the epithelial turnover and induces higher number of refractive structures in the stroma. A previous study performed at our laboratory demonstrated a reduced number of superficial and an increased number of basal epithelial cells indicating a higher epithelial turnover in DE conditions and high metabolic activity [4]. 

 The increased number of cellular bodies found in the stroma corresponds to inflammatory cell infiltration and activated keratocytes (fibroblasts and myofibroblasts) that may have been activated by the epithelial injury. Eight to 24 hours after an epithelial injury, chemokines released by the epithelium and produced by the keratocytes in response to cytokine stimulation attract inflammatory cells such as polimorphonuclear leukocytes, macrophages/monocytes, and T cells into the stroma from the limbal vessels and possibly from the tears [6]. The main function of these cells is the phagocytosis of apoptotic bodies and residual necrotic cellular fragments. The DE may cause additional epithelial cell damage that increases the liberation of proinflammatory cytokines such as IL-1, TNF-*α*, and TGF-*β* inducing an increased activation of keratocytes in the corneal stroma. Many of the proinflammatory chemokines that induce a greater influx of inflammatory cells such as granulocyte colony-stimulating factor (G-CSF), neutrophil activating peptide (ENA-78), monocyte-derived neutrophil chemotactic factor (MDNCF), and monocyte chemotactic and activating factor (MCAF) have an upregulated expression in activated keratocytes [7]. 

A very important inflammatory mediator released after corneal injury is PAF [8]. The action of PAF is mediated by specific receptors whose expression is enhanced after injury [9]. During PRK, the injured epithelium releases PAF in the area adjacent to the anterior stroma and in the lamellar interface and triggers an inflammatory reaction and induces keratocyte apoptosis and activation of distant keratocytes and transformation to myofibroblasts. Keratocyte apoptosis that occurs in response to the epithelial injury associated with PRK is one of the main initiators of the corneal wound healing response [10, 11]. Our results suggest that PAF is involved in the inflammatory cell infiltration and expression of inflammatory cytokines that follow PRK under DE. Treatment with PAF receptor antagonist (LAU-0901) resulted in reduced expression of inflammatory mediators such as COX-2 and ASE in the corneal stroma compared with vehicle. 

 Elevated expression of ASE and Cox-2 in mice after PRK and exposed to a desiccating environment may play a role in the healing response. It is possible that the monocytes use the arginine-ornithine pathway to repair the damage caused by the inflammatory response after surgery. Expression of NO synthase creates a cytotoxic environment that may be important in the early phase of wound healing [12]. In the early stages arginine can be metabolized by inflammatory cells through the oxidative 1-arginine deiminase that results in the formation of citrulline and reactive nitrogen intermediates [13]. These compounds may mediate some of the events characteristic of early inflammation. As wound healing progresses, increased ASE activity increases the catabolism of arginine and produces an environment favorable for fibroblast replication and collagen production.

 The inflammatory response induced by PRK produces keratocyte apoptosis. The keratocytes adjacent in the tissue proliferate and migrate to the injured site and undergo fibroblast and myofibroblast transformation. The myofibroblast—a specialized contractile fibroblast—has an important role in establishing tension during wound healing and pathological contracture. Differentiated myofibroblasts lay down collagen, chondroitin sulfate, and other ECM components and produce proteases. Myofibroblasts express PAF receptors [14] and this cytokine induces the synthesis of MMPs [15]. Once the wound closes and the basal membrane regenerates, the myofibroblasts disappear, probably by apoptosis. However, if they persist and continue to remodel the ECM, the process results in connective tissue contraction, scar formation, induced irregular astigmatism and abnormal epithelial healing, and possibly stromal necrosis and melting. Our results demonstrated that blocking the inflammatory cascade with LAU-0901 during the first postoperatively after PRK reduces degeneration of myofibroblasts in the corneal stroma which may improve the predictability of the refractive results after surgery.

 Because the corneal wound healing response is so complex, it is difficult to intervene at more advanced steps in the process. Instead, blocking the initial inflammatory cell infiltration response is the most effective approach. The interaction between the overlying epithelium and the keratocyte cells and the continuing communication between the two are key factors that lead to the successful outcome of the healing response. Manipulating the initial inflammatory cell infiltration with LAU-0901 may affect the corneal wound healing response and may have implications in the predictability and stability of the refractive results after PRK.
